# Fang Filling

**Published:** 1860-08

**Authors:** A. A. Blount

**Affiliations:** Springfield


### "FANG FILLING."

Read at a meeting of the Mad River Valley Society,

BY A. A. BLOUNT, M. D.

"FANG FILLING," till quite recently, was considered an unsafe and hazardous operation. The principal objections that have been urged against the practice, are, that in proportion as you destroy the vitality of the nerve, you render the tooth more liable to violent attacks of inflammation, and, consequently, a constant source of irritation to the surrounding parts. It is maintained by many that, notwithstanding the tooth may not give rise to alveolar abscess, it is, nevertheless, a constant source of irritation to the contiguous living parts, and that its removal is the only remedy. The success of those who formerly attempted the experiment of retaining the teeth, after the destruction of the living membrane, fully justified the conclusion, and, in almost every instance, the operation was a total failure, unless some opening was left, in the filling or tooth, to admit of the escape of the secretions and gases.

This want of success was soon traced to the proper source; and a new field was opened to the enterprising Dentist. The fangs were then freed from the *dead membrane* and filled; and success was the result of almost every experiment.

It was argued that, should there be a secretion of matter at the root, the absorbents would carry it off as fast as it was formed, and that there would be sufficient vitality supplied to the tooth by the investing membrane.

By the repeated experiments of those who have been foremost in the ranks, such a degree of perfection has been attained that, although a tooth may have been suppurating for years, and alveolar abscess may have been of frequent occurrence, leaving fistulous openings in the gums, it can be restored to perfect health and usefulness.

The first step toward the treatment of an exposed nerve, is to consider the best means of accomplishing its destruction and removal. There are several means, and much diversity of opinion as regards the most efficient, and the one least liable to produce disastrous consequences. The two which seem most in favor are " arsenic," and extirpation with an instrument. The nerves in the incisor teeth can be more readily extirpated with a small, untempered instrument, filed flat, and twisted in the shape of a screw, at the point. Pass this into the cavity, giving it a slight rotary motion, and the nerve becomes entangled with the instrument, and frequently may be brought away entire, with but a moment's pain.

In using arsenic, care should be taken not to apply too much; as there is no doubt that the frequent failures of many in the use of it, are attributable to that cause.

If we will take pains to freely expose the nerve, so that we can apply the arsenic in contact with it, we will more readily accomplish its destruction, and be less liable to disastrous results, from the absorption of the poison. The smallest quantity, therefore, say the fortieth or fiftieth part of a grain, will be sufficient. (I very frequently use no more than will adhere to the point of a small instrument, dipped into it, with which I touch the nerve.)

This should, in no instance, be left in the tooth longer than twenty-four hours,—in young persons, eight to ten hours is sufficient.

After removing the application, take a chisel or drill, and expose the entire pulp cavity, that you may more readily have access to the fang. We have now arrived at a point where the least neglect will cause a total failure. It is *absolutely necessary* that *every portion* of the *nerve* should be *removed*, and the *cavity* freed from any *foreign substance* whatever. This precaution is indispensable to a successful result.

There is much difficulty, in most cases, in extirpating the nerves in the buccal fangs of the upper molars,—they are generally very small, and will seldom admit the smallest sized broach. To obviate this difficulty, take a very sharp, four-sided reamer, and ream them to the end of the fang. The cavity should be syringed with water; and after drying well, introduce a pledget of cotton, saturated with creosote, seal up, and let remain two or three days. After removing, take a small broach, flattened at the point, turned up at right angles, and made very sharp. With this, we can free the cavity from any foreign substance that may be left remaining in it. It is then, provided there is no inflammation or soreness, ready for the filling.

We have, so far, only spoken of cases of the most simple character, and now come to those more complicated—with alveolar abscess, and fistulous openings in the gums. These cases will require more care and attention to produce the desired results. And, in order to be successful in the treatment of them, we should have a sound constitution to aid us in our efforts, or we will be likely to fail.

The first step is to make an opening in the crown, sufficiently large to expose the fangs to view. This can be easily accomplished with chisel and drill. Cleanse them thoroughly with excavator and syringe, and apply either creosote, chloride of zinc, or nitrate of silver. Seal up, and let remain from one to three days. If there be much discharge of matter, the application should be frequent; and, as the discharge diminishes, lengthen the time between applications.

To be enabled more readily to reach the point of the fang, take a piece of floss silk, say an inch or two long, saturate with the remedy, and with a broach you can introduce it as far up as desired, taking care, however, to leave one end hanging out, to enable you to withdraw it easily, otherwise you may have some difficulty. Continue the treatment till the tooth exhibits no signs of inflammation, or soreness from pressure; and you can then fill it with perfect confidence as to the result.

Should there be a fistulous opening in the gum, make an incision with gum lancet across it; introduce floss silk, saturated with tannin or tinct. of iodine, and keep it open till after the tooth is filled, and then allow it to heal.

Mr. B., of a robust and healthy constitution, called to have the right superior molar extracted, which had been ulcerating for *four years*, and discharging through a fistula in the palatal arch. The tooth being too valuable to think of extracting, I commenced the treatment by cutting away all the decayed and soft parts, exposing the cavities in the fangs to view, cleansed them thoroughly with the syringe, freed them of the dead and ulcerating pulp, and, with reamer and drills, enlarged the cavities as much as was practicable, to enable me more readily to reach the points of the fangs; with a broach, I introduced into each floss silk, saturated with creosote. This was renewed every third or fourth day, till there was no discharge perceptible.

With a gum lancet I made an incision across the fistula, reaching to the alveolus at the point of the fang, which I found somewhat diseased. With a strong excavator I broke away the diseased bone, and introduced a pledget of cotton, saturated with tinct. of iodine. The fistula was kept open till after the tooth was filled, and then allowed to heal, which it did in a few days, leaving no trace of its former location.

It sometimes happens, after the destruction of the lining membrane, the tooth becomes several shades darker. It is desirable, before filling, to restore the natural color, especially if it be an incisor,—otherwise it would present an unsightly appearance.

To effect this desirable object, I have been in the habit of using a solution of "Chloride of Soda and Lime." Introduce cotton, saturated with the mixture, seal up, and let remain twenty-four hours. One application will generally be sufficient.

In filling the fangs, I use soft and adhesive foil, which I prepare by cutting the sheet into strips from a half inch, to an inch in width, rolling it, and cutting into various sized pellets or blocks. Take one of the smallest sized pellets on the point of a plugger, dip it into creosote, introduce it into the cavity carefully, and slowly press it up, that the air may escape. Then dry the cavity well, and finish filling.

After filling the fangs, let the tooth remain a day or two, and fill the crown.

If we are permitted to judge from the agitation of the subject in the various Dental periodicals of the day, there seems to be quite a controversy between the advocates of the two methods of filling teeth of this character. One party maintaining that it is indispensable to the preservation of the tooth to fill the fangs, while, on the other hand, it is claimed to be unnecessary, and that filling the crown only is equally as successful.

I am not prepared to speak, from any great amount of experience, of the latter method, having filled but very few teeth without filling the fangs.

But I trust I will not trespass on your time and patience, by giving you my views of the method adopted by our eminent brother and worthy President, Dr. Watt, who was, I believe, the first to make public this method of filling teeth. In preparing the fangs, (we all know in order to be successful,) the nerve *must* be removed to the end of the cavity, or just where it passes through the periosteum that covers the fang, where it will either heal by first intention, or by suppuration and granulation. Beyond that point, we can not remove the nerve, without wounding the soft parts in such a manner that violent inflammation supervenes, producing suppuration, and in all probability, the death of the periosteum. And, should we fail to remove the nerve to that point, we are also aware that the operation would be a failure. Now, if we are successful in removing it, and it heals and closes up the end of the fang, what is the use of filling it? There is no chance of any secretions entering the cavity; and the tooth is just as safe as though it were filled with gold.

If we will adopt the precaution never to fill a tooth till we are assured that the nerve has healed, we will never experience the mortification of a failure, and I am of the opinion whether we fill the fangs or not, the operation will be equally successful.

SPRINGFIELD, July 2d, 1860.

---

